# Simultaneous Determination of *Fucoxanthin* and Its Deacetylated Metabolite Fucoxanthinol in Rat Plasma by Liquid Chromatography-Tandem Mass Spectrometry

**DOI:** 10.3390/md13106521

**Published:** 2015-10-23

**Authors:** Yiping Zhang, Hao Wu, Hongmei Wen, Hua Fang, Zhuan Hong, Ruizao Yi, Rui Liu

**Affiliations:** 1 Third Institute of Oceanophy Sgratate Oceanic Administration, Xiamen 361005, Fujian, China; E-Mails: ypzhang@tio.org.cn (Y.Z.); fhua@tio.org.cn (H.F.); zhong@tio.org.cn (Z.H.); 2 College of Pharmacy, Nanjing University of Chinese Medicine, Nanjing 210023, Jiangsu, China; E-Mails: njwenhm@126.com (H.W.); cpulr@126.com (R.L.); 3 Fujian Collaborative Innovation Center for Exploitation and Utilization of Marine Biological Resources, Xiamen 361005, Fujian, China

**Keywords:** fucoxanthin, fucoxanthinol, metabolite, LC-MS/MS, pharmacokinetics

## Abstract

Fucoxanthin and its deacetylated metabolite fucoxanthinol are two major carotenoids that have been confirmed to possess various pharmacological properties. In the present study, fucoxanthinol was identified as the deacetylated metabolite of fucoxanthin, after intravenous (i.v.) and intragastric gavage (i.g.) administration to rats at doses of 2 and 65 mg/kg, respectively, by liquid chromatography-tandem mass spectrometric (LC-MS/MS) analysis. Next, an accurate and precise LC-MS/MS method was developed to quantitatively determine fucoxanthin and fucoxanthinol in rat plasma. Plasma samples were resolved by LC-MS/MS on a reverse-phase SB-C18 column that was equilibrated and eluted with acetonitrile (A)/aqueous 0.1% formic acid (B; 92/8, *v*/*v*) at a flow rate of 0.5 mL/min. Analytes were monitored by multiple-reaction monitoring (MRM) under positive electrospray ionization mode. The precursor/product transitions (*m*/*z*) were 659.3→109.0 for fucoxanthin, 617.2→109.0 for fucoxanthinol, and 429.4→313.2 for the internal standard (IS). Calibration curves for fucoxanthin and fucoxanthinol were linear over concentrations ranging from 1.53 to 720 and 1.17 to 600 ng/mL, respectively. The inter- and intraday accuracy and precision were within ±15%. The method was applied successfully in a pharmacokinetic study and the resulting oral fucoxanthin bioavailability calculated.

## 1. Introduction

**Figure 1:**
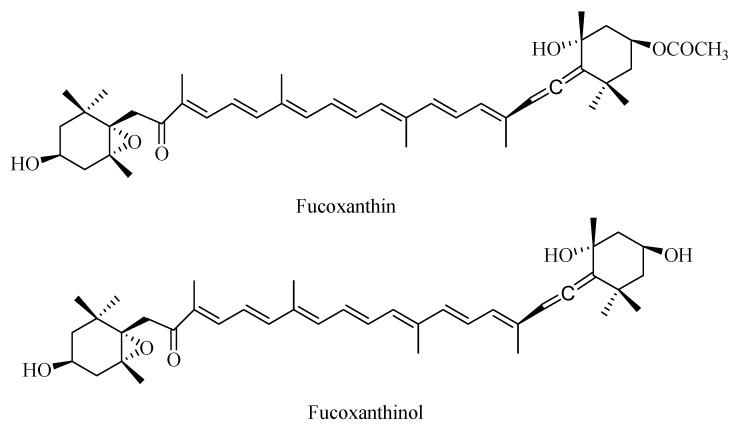
Structures of fucoxanthin and fucoxanthinol.

Fucoxanthin is one of the most abundant carotenoids, representing more than 10% of the estimated total natural carotenoid production, especially in the marine environment [1]. Containing an unusual allenic bond and 5,6-monoepoxide (Figure 1), this uniquely structured compound also possesses various physiological activities. It has been reported that fucoxanthin exhibits biological functions, including anticancer, antihypertensive, anti-inflammatory, antioxidant, and anti-obesity effects; antidiabetic activity; and hepatoprotective effects [2,3,4,5,6,7,8,9,10,11,12,13]. Fucoxanthinol, the deacetylated product of fucoxanthin, has physiological activities similar to fucoxanthin [14,15,16,17,18]. Investigations into digestion, absorption, and metabolism of dietary fucoxanthin have suggested that fucoxanthin might be hydrolyzed to fucoxanthinol in the gastrointestinal tract by digestive enzymes, such as lipase and cholesterol esterase. Subsequently, fucoxanthinol is absorbed in intestinal cells then converted to amarouciaxanthin A in the liver [14,19]. Tatsuya *et al.* have shown that fucoxanthinol is detected in mouse plasma 1 h after intubation of 40 nmol of fucoxanthin [20]. These results indicate that dietary fucoxanthin is incorporated as fucoxanthinol, the deacetylated form, from the digestive tract into the blood circulation in mammals. However, fucoxanthin does not appear in mouse plasma after fucoxanthin ingestion [20], indicating the absence in mice of absorption, fucoxanthinol and processing of oral fucoxanthin. Takashi *et al*. have also reported the distribution and accumulation of orally-administered fucoxanthin and its subsequent metabolites amarouciaxanthin A in the plasma, erythrocytes, liver, lung, kidney, heart, spleen, and adipose tissue [21]. In their study, animals were killed 1, 2, 3, 4, 6, 9, 12, 24, 48, and 72 h after fucoxanthin administration, which could not eliminate individual subject differences in fucoxanthin absorption and processing. There have been some reports regarding absorption of orally administered fucoxanthin, but intravenous (i.v.) administered fucoxanthin as well as its pharmacokinetic parameters are little understood.

For further development and rational use of fucoxanthin, it was considered important to develop a method for quantitative analysis of bioactive fucoxanthin in plasma samples. In the present investigation, a simple and rapid liquid chromatography-tandem mass spectrometric (LC-MS/MS) analytical method was established for simultaneous quantification of both fucoxanthin and fucoxanthinol from rat plasma, with hydroxyprogesterone caproate employed as an internal standard (IS). This method proved to be sensitive and selective, with a wide detection range and low detection limit. In the present study, rats were administered intragastric (i.g.) and i.v. fucoxanthin and the pharmacokinetic parameters of fucoxanthin and fucoxanthinol observed. This study might provide preliminary pharmacokinetic information regarding fucoxanthin and fucoxanthinol for future pharmacology studies.

## 2. Results

### 2.1. Identification of Fucoxanthin and Fucoxanthinol by LC-MS/MS

**Figure 2:**
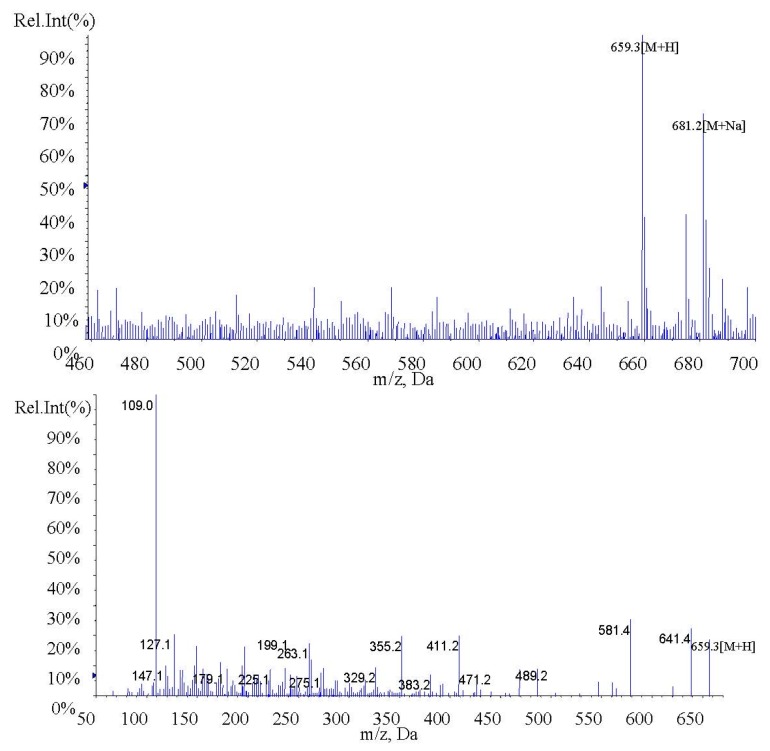
MS1 and MS2 of fucoxanthin.

**Figure 3:**
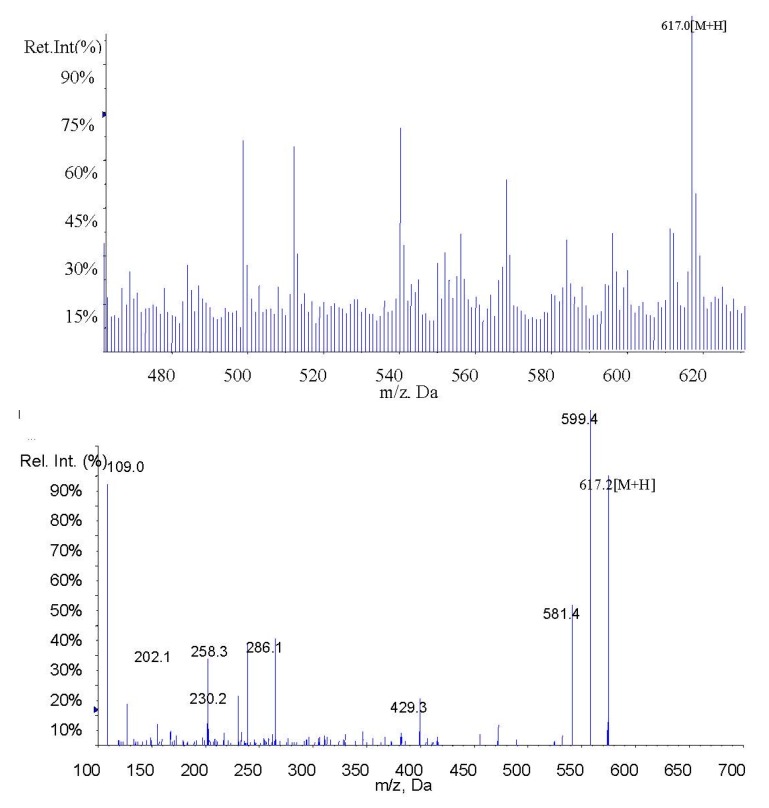
MS1 and MS2 of fucoxanthinol.

**Figure 4:**
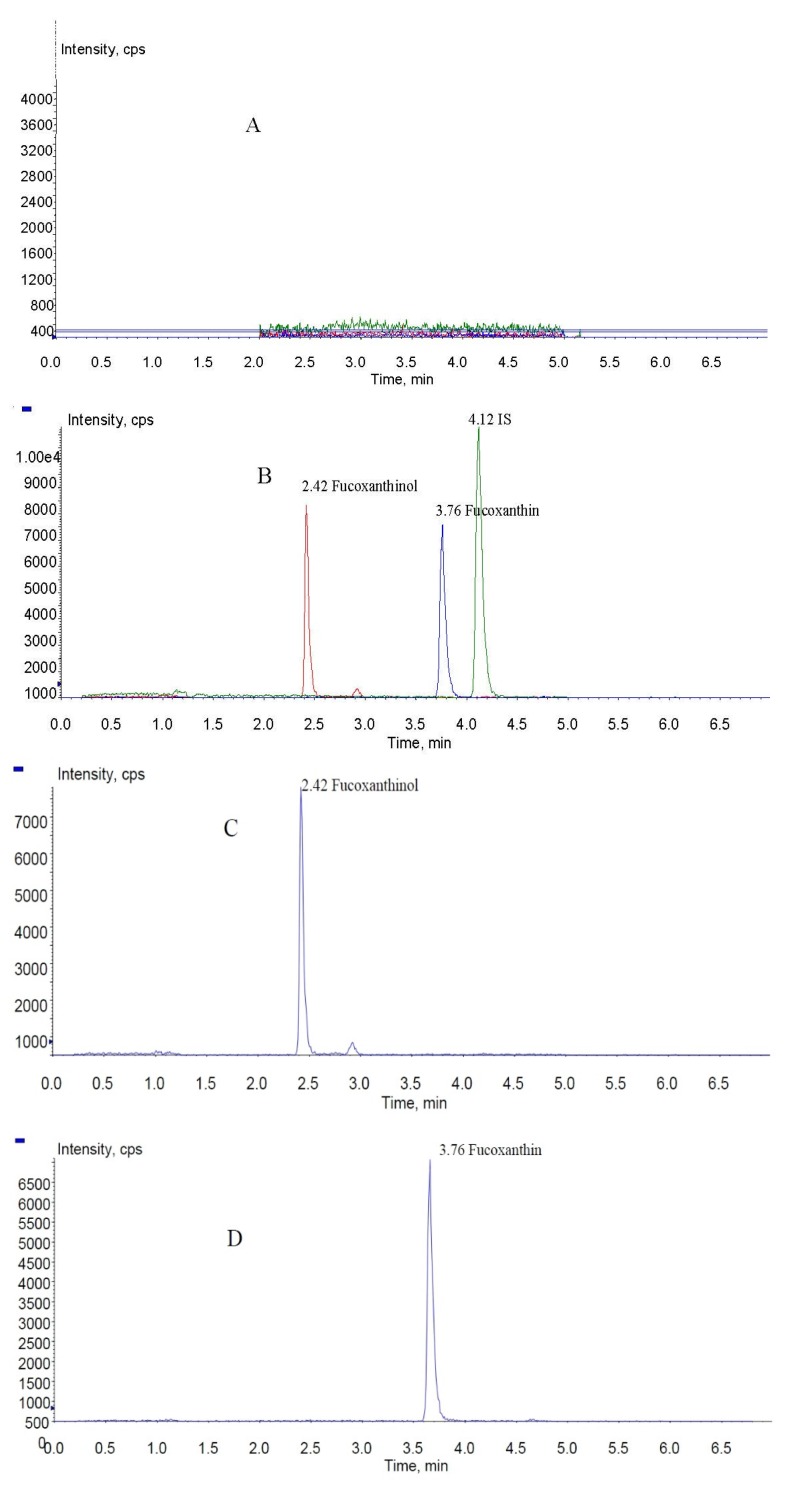

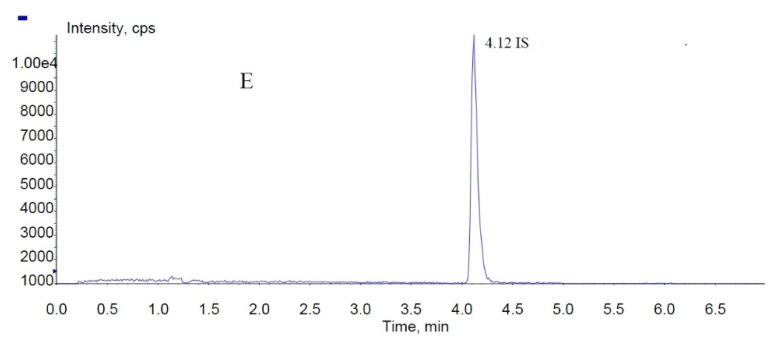
MRM chromatograms of fucoxanthin, fucoxanthinol, and IS (internal standard): (**A**) blank rat plasma sample; (**B**) total ion chromatogram spiked with fucoxanthin, fucoxanthinol, and IS; and (**C**–**E**) fucoxanthinol, fucoxanthin, and IS MRM (multiple-reaction monitoring) chromatograms, respectively.

In the present study, fucoxanthin and its deacetylated metabolite fucoxanthinol were identified by liquid chromatography-tandem mass spectrometry (LC-MS/MS). Detection was performed in the positive ion mode and conditions for fucoxanthin and fucoxanthinol detection optimized using standards. Daughter ions obtained from protonated molecular ions of fucoxanthin ([M + H]-659.3) and fucoxanthinol ([M + H]-617.2) included three main ions from each compound at *m*/*z* 109.0, 581.4, and 641.4 (Figure 2) and at *m*/*z* 109.0, 581.4, and 599.4, respectively (Figure 3). Identification was developed by monitoring *m*/*z* transitions of 659.3→109.0 and 659.3→581.4 for fucoxanthin and 617.2→109.0, 617.2→581.4 for fucoxanthinol; the corresponding parameters are shown in Table 1. The peak area ratio of the two coupled transitions for each analyte were matched to a standard. Indices for identification were also generated, yielding values for fucoxanthin and fucoxanthinol at 3.76 ± 0.10 (peak area ratio of transitions *m*/*z* 659.3→109.0 to 659→581.4) and 2.42 ± 0.10 (peak area ratio of transitions *m*/*z* 617.2→109.0 to *m*/*z* 617.2→581.4), respectively. The total chromatography time was 7.0 min and retention times for fucoxanthin, fucoxanthinol, and IS were 3.76, 2.42, and 4.12 min, respectively (Figure 4). Both fucoxanthin and fucoxanthinol were detected in samples after i.v. and i.g. administration.

**Table 1:** ESI-MS/MS parameters for fucoxanthin, fucoxanthinol, and IS (internal standard).

Analyte	Precursor Ion (*m*/*z*)	Daughter Ion (*m*/*z*)	Dwell Time (s)	DP (V)	EP (V)	CE (V)	CXP (V)
Fucoxanthin	659.3	109.0, 581.4, 641.4	0.2	48	10	28	18
Fucoxanthinol	617.2	109.0, 581.4, 599.4	0.2	15	11	28	8
IS	429.4	313.2	0.2	60	10	37	13

### 2.2. Method Validation

#### 2.2.1. Selectivity and Specificity

Selectivity and specificity were assessed by comparing chromatograms of six different batches of blank rat plasma samples to corresponding spiked plasma samples. No endogenous substances were observed to interfere with fucoxanthin, fucoxanthinol, and IS in any samples. Specificity was verified by comparing retention times of fucoxanthinol, fucoxanthin, and IS (2.42, 3.76, and 4.12 min, respectively) in quality control (QC) samples (*n* = 6), which showed differences of less than 2%.

#### 2.2.2. Calibration Curve Linearity, Lower Limit of Quantification, and Limit of Detection

Standard curves were established by plotting the ratios of chromatogram peak areas of fucoxanthin and fucoxanthinol to those of IS. The curves showed correlation coefficients of >0.993 and exhibited good linearity over concentration ranges of 1.53–720 and 1.17–600 ng/mL for fucoxanthin and fucoxanthinol, respectively. Typical calibration equations were *y* = 0.000677*x* + 0.000749 (*R* = 0.994) for fucoxanthin, and *y* = 0.000582*x* + 0.000856 (*R* = 0.993) for fucoxanthinol, where *y* represents the peak area ratio of an analyte to that of IS, and *x* represents an analyte concentration. The lower limits of quantification (LLOQ) for both fucoxanthin and fucoxanthinol were defined as 1.53 and 1.17 ng/mL, respectively, based on a signal-to-noise ratio (S/N) of 10. The limit of detection (LOD) was estimated to be <0.77 and <0.59 ng/mL, respectively, based on an S/N of 3.

#### 2.2.3. Accuracy and Precision

The method’s accuracy and precision are summarized in Table 2, following criteria for biological sample analysis according to U.S. Food and Drug Administration (FDA) guidelines. Accuracy was required to be within ±15% (20% for LLOQ) and precision not to exceed ±15% (20% for LLOQ). The present results suggested that the method was accurate and precise for simultaneous analysis of fucoxanthin and fucoxanthinol in rat plasma samples.

**Table 2:** Accuracy and precision for determination of fucoxanthin and fucoxanthinol in plasma samples (*n* = 5).

Analyte	Concentration (ng/mL)	Intraday			Interday		
		Mean ± SD (ng/mL)	Precision (%)	Accuracy (%)	Mean ± SD (ng/mL)	Precision (%)	Accuracy (%)
Fucoxanthin	11.25	12.63 ± 0.25	1.99	111.80	11.83 ± 0.84	7.09	104.72
	90	96.80 ± 4.54	4.69	107.56	80.87 ± 2.34	2.89	89.96
	360	378.67 ± 21.23	5.74	105.19	312.00 ± 12.29	12.29	86.67
Fucoxanthinol	9.4	10.42 ± 0.65	6.26	110.89	9.91 ± 0.48	4.84	105.46
	75	81.93 ± 4.55	5.56	109.24	68.77 ± 0.58	0.84	91.69
	300	317.00 ± 8.72	2.75	105.67	265.33 ± 4.51	1.7	88.44

#### 2.2.4. Recovery and Matrix Effects

The recoveries of fucoxanthin and fucoxanthinol spiked into rat plasma were determined at three QC concentrations. The recoveries of fucoxanthin were 101.00% ± 3.61%, 96.73% ± 7.62%, and 92.90% ± 2.91% (*n* = 3) at concentrations of 12.25, 90, and 360 ng/mL and those of fucoxanthinol were 91.58% ± 1.84%, 84.76% ± 2.44%, and 88.43% ± 1.57% (*n* = 3) at concentrations of 9.4, 75, and 300 ng/mL, respectively (Table 3). Matrix effects in the present study were investigated by a post-extraction spike method. The peak area of a standard analyte spiked into blank plasma was compared with the corresponding peak area obtained by directly injecting the standard analyte in the mobile phase at concentrations of 12.25, 90, and 360 ng/mL for fucoxanthin and 9.4, 75, and 300 ng/mL for fucoxanthinol, all performed in triplicate. In terms of matrix effect, all the ratios defined above were within acceptable limits (85.90%–98.44%). No significant matrix effect for fucoxanthin and fucoxanthinol was observed, indicating that ion suppression or enhancement from plasma components was negligible for this method.

**Table 3:** Recovery and matrix effects of fucoxanthin and fucoxanthinol in plasma samples (*n* = 5).

Analyte	Concentration (ng/mL)	Recovery		Matrix Effects	
		Mean ± SD (ng/mL)	RSD (%)	Mean ± SD (ng/mL)	RSD (%)
Fucoxanthin	12.25	101.00 ± 3.61	3.61	86.92 ± 14.34	16.50
	90.0	96.73 ± 7.62	7.88	87.71 ± 9.91	11.29
	360	92.90 ± 2.91	3.14	98.44 ± 9.32	9.46
Fucoxanthinol	9.4	91.58 ± 1.84	2.01	85.90 ± 14.47	16.84
	75	84.76 ± 2.44	2.88	87.59 ± 7.60	8.67
	300	88.43 ± 1.57	1.78	95.08 ± 7.51	7.90

RSD, relative standard deviation.

#### 2.2.5. Stability

The stability of fucoxanthin and fucoxanthinol were evaluated as described in the experimental section. Results from all stability tests indicated that these analytes were stable under routine laboratory conditions (Table 4). The method was therefore judged to be applicable for routine analyses.

**Table 4:** Stability of fucoxanthin and fucoxanthinol (*n* = 6).

Storage Condition (−80 °C)	Fucoxanthin			Fucoxanthinol		
	Concentration (ng/mL)	Accuracy (%)	RSD (%)	Concentration (ng/mL)	Accuracy (%)	RSD (%)
1 freeze-thaw cycle	12.25	82.55	4.03	9.4	99.60	4.83
	90.0	87.40	1.78	75	89.05	5.00
	360	80.35	3.78	300	89.45	4.03
2 freeze-thaw cycles	12.25	102.20	6.64	9.4	101.90	12.63
	90.0	80.35	0.44	75	91.30	8.83
	360	86.75	3.67	300	91.75	8.25
3 freeze-thaw cycles	12.25	113.0	7.51	9.4	118.0	5.52
	90.0	90.55	3.36	75	99.70	6.10
	360	85.95	1.89	300	98.60	0.14

### 2.3. Pharmacokinetic Studies of Fucoxanthin and Fucoxanthinol

#### 2.3.1. Studies of i.v. Fucoxanthin Administration

**Figure 5:**
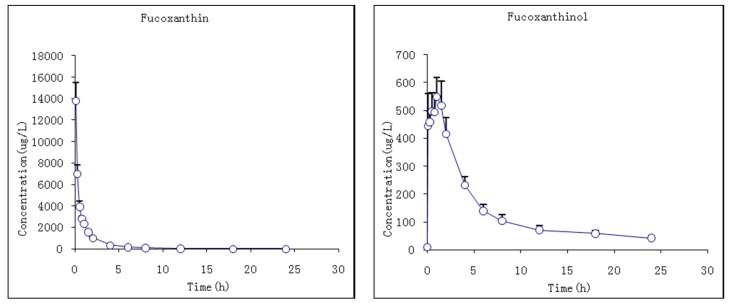
Mean plasma concentration–time profiles of fucoxanthin and fucoxanthinol in rats following i.v. (intravenous) administration of 2 mg/kg fucoxanthin (*n* = 6; circle and bar, mean and ± SD).

The pharmacokinetic profiles of fucoxanthin and fucoxanthinol were investigated by the described method following a single i.v. injection of 2 mg/kg fucoxanthin to rats. The mean plasma concentration–time profiles of fucoxanthin and fucoxanthinol showed that fucoxanthin concentrations declined sharply by 120 min after dosing, decreasing from 13,783 ± 1544 to 1011 ± 77 ng/mL, and decreased more slowly thereafter (Figure 5). Meanwhile, the metabolite fucoxanthinol was detected 5 min after fucoxanthin dosing, which suggested that fucoxanthin was converted quickly to fucoxanthinol in rat plasma. The T_max_ of fucoxanthinol was 1 ± 0.4 h, which suggested that the conversion rate of fucoxanthin to fucoxanthinol was more rapid than the fucoxanthinol eliminate rate at 1 h. The C_max_ of fucoxanthinol was 598.2 ± 64.8 ng/mL, which was much lower than that of fucoxanthin. The pharmacokinetic parameters of fucoxanthin and fucoxanthinol were calculated and are summarized in Table 5. After i.v. fucoxanthin administration, the mean values of systemic clearance were 0.2 and 0.5 L/h/kg for fucoxanthin and fucoxanthinol, respectively. The AUC_0–∞_ value for fucoxanthin (9871.0 μg·h/L) was about 2.5-fold larger than that for fucoxanthinol (3954.7 μg·h/L), indicating that most fucoxanthin was quickly transformed into fucoxanthinol in these rats and, thus, its major metabolite fucoxanthinol appeared to play an important role in the subsequent pharmacological action of i.v. administered fucoxanthin.

**Table 5:** Pharmacokinetic parameters of fucoxanthin and fucoxanthinol in rats after i.v. (intravenous) fucoxanthin administration at 2 mg/kg body wt.

Parameters	Unit	Fucoxanthin		Fucoxanthinol	
		Mean	S.D.	Mean	S.D.
C_max_	μg/L			598.2	64.8
T_max_	h			1.0	0.4
AUC_0–*t*_	μg·h/L	9861.8	749.0	3260.6	326.1
AUC_0–∞_	μg·h/L	9871.0	746.9	3954.7	354.8
*t*_1/2_	h	2.3	0.8	11.9	2.2
CL	L/h/kg	0.2	0.0	0.5	0.0
Vd	L/kg	0.7	0.3	8.8	2.0

CL, total body clearance and Vd, steady state apparent volume of distribution.

#### 2.3.2. Studies of i.g. Fucoxanthin Administration

**Figure 6:**
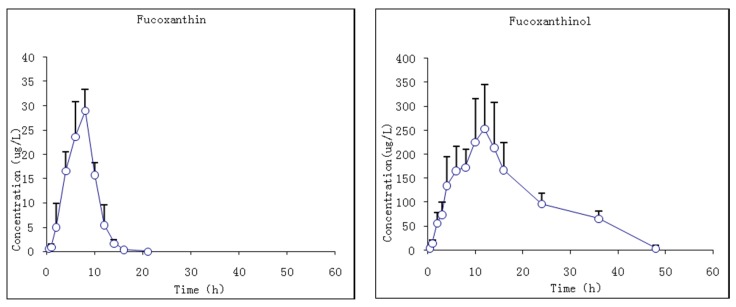
Mean plasma concentration–time profiles of fucoxanthin and fucoxanthinol in rats following i.g. (intragastric gavage) fucoxanthin administration at 65 mg/kg body wt of fucoxanthin (*n* = 6; circle and bar, mean and ±SD).

The pharmacokinetic profiles of fucoxanthin and fucoxanthinol were investigated by the described method following a single i.g. dose at 65 mg/kg body wt of fucoxanthin to rats. The mean plasma concentration–time profiles of fucoxanthin and fucoxanthinol showed that fucoxanthin was absorbed slowly by rats such that it was detected in plasma only 0.5 h after administration and rose slowly thereafter (Figure 6). The T_max_ of fucoxanthin was 7.7 ± 0.8 h and the C_max_ 29.1 ± 4.4 μg/L. Fucoxanthin was eliminated rapidly, having a *t*_1/2_ of 1.2 ± 0.6 h. Meanwhile, fucoxanthinol was detected after fucoxanthin dosing, with a C_max_ of 263.3 ± 93.3 μg/L, which is nearly 10-fold that of fucoxanthin. This suggested that fucoxanthin was largely converted to fucoxanthinol in rat intestine, and then absorbed. The T_max_ of fucoxanthinol was 11 ± 1.1 h and its elimination rate slower then fucoxanthin, the former having a *t*_1/2_ of 9.3 ± 5.8 h. The pharmacokinetic parameters of fucoxanthin and fucoxanthinol were calculated and are summarized in Table 6. These results suggested that parent drug elimination was substantially faster than that of fucoxanthinol in rats with the same i.v. administration fucoxanthin. The AUC_0–t_ value for fucoxanthin was much lower than that for metabolite fucoxanthinol (191.1 and 5017.6 μg·h/L, respectively), indicating that fucoxanthinol was absorbed more easily than fucoxanthin in rats and supported the earlier conclusion from i.v. studies that fucoxanthinol appeared to play an important pharmacological role.

**Table 6:** Pharmacokinetic parameters of fucoxanthin and fucoxanthinol in rats after i.g. (intragastric gavage) fucoxanthin administration at 65 mg/kg body wt.

Parameters	Unit	Fucoxanthin		Fucoxanthinol	
		Mean	S.D.	Mean	S.D.
C_max_	μg/L	29.1	4.4	263.3	93.9
T_max_	h	7.7	0.8	11	1.1
AUC_0–t_	μg·h/L	191.1	39.6	5017.6	1371.0
AUC_0–∞_	μg·h/L	195.1	42.2	5304.2	1266.9
t_1/2_	h	1.2	0.6	9.3	5.8
CL	L/h/kg	347.8	81.8	12.8	3.1
V_d_	L/kg	550.2	226.1	181.5	142.4

## 3. Discussion

Diverse carotenoids with different chemical structures exist in edible plants in significant amounts. Fucoxanthin is one of the major xanthophylls in brown algae chloroplasts and possesses as well unique chemical features as pharmacological effects. Fucoxanthin is able to inhibit expression of the *N*-myc oncogene, cell cycle progression in human neuroblastoma cell lines, GOTO cells, *N*-ethyl-*N′*-nitro-*N*-nitrosoguanidine-induced mouse duodenal carcinogenesis, and the growth of human promyelocytic leukemia HL-60 cell by apoptosis induction [22]. Recent studies have suggested that dietary fucoxanthin is hydrolyzed to fucoxanthinolin the gastrointestinal tract by digestive enzymes, such as lipase and cholesterol esterase, absorbed by intestinal cells, and converted to amarouciaxanthin A in the liver. Fucoxanthinol has been detectable at 0.8 pmol/mL in human plasma after a daily intake of stir-fried wakame seaweed (6 g dry wt) including 6.1 mg (9.26 μmol) of fucoxanthin for 1 week [21]. These reports illustrate the absorption and metabolism mechanisms of dietary fucoxanthin but did not explain fucoxanthin bioavailability and other pharmacokinetic parameters.

Takashi *et al.* have reported that, after a single oral administration of 160 nmol of fucoxanthin, fucoxanthinol was detectable in all specimens but fucoxanthin was not [21]. In the present study, greater proportions of fucoxanthin (65 mg/kg) were orally administered to rats, after which fucoxanthin was detected, albeit at very low plasma concentrations. Fucoxanthin’s oral bioavailability was very low, at just 0.06%, calculated following a previously published equation [23]. Detection of fucoxanthin and fucoxanthinol after i.v. and i.g. administrations reflected that fucoxanthin might have been metabolized to fucoxanthinol, but it might also have been further metabolized to other forms. There might be three reasons for fucoxanthin’s low bioavailability. First, fucoxanthin possesses low solubility in water and low cell penetrability. Second, fucoxanthin can be rapidly metabolized to fucoxanthinol in plasma. Finally, fucoxanthin can be metabolized to fucoxanthinol in the intestine. It is expected that the present results will be helpful for improving clinical therapeutic efficacy and further pharmacological studies of fucoxanthin and fucoxanthinol.

## 4. Experimental Section

### 4.1. Chemicals and Reagents

Fucoxanthin and fucoxanthinol were prepared and identified by UV, MS, and NMR spectroscopies in our laboratory (purity ≥99%). Hydroxyprogesterone caproate (purity ≥98%), the IS, was purchased from the National Institutes for Food and Drug Control (Beijing, China). HPLC-grade acetonitrile and methanol were obtained from Merck KGaA (Darmstadt, Germany). HPLC-grade formic acid was obtained from Roe Scientific Inc. (Powell, OH, USA). Ultrapure water was produced by a Millipore Milli-Q system (Millipore Corp., Billerica, MA, USA). All other reagents or solvents were commercially available and reagent grade. Blank rat plasma was collected from healthy male Sprague-Dawley rats weighing 200 ± 20 g (Laboratory Animal Center of Xiamen University, Xiamen, China).

### 4.2. Chromatographic and Mass Spectrometric Conditions

#### 4.2.1. Liquid Chromatography Conditions

Analyte separations were performed on an Acquity UPLC-1290 system (Agilent Corp., Milford, MA, USA) using an SB-C18 column (150 × 2.1 mm, 2.1 mm i.d.; Agilent Technologies, Inc., Santa Clara, CA, USA) maintained at 35 °C. The mobile phase was composed of acetonitrile (A) and aqueous 0.1% formic acid (B; 92/8, *v*/*v*) at a flow rate of 0.5 mL/min and the injection volume was 5 μL.

#### 4.2.2. Mass Spectrometric Conditions

Identification of fucoxanthin and fucoxanthinol in plasma samples was conducted using an AB 5500 Q-trap LC-MS/MS (ABSCIEX, Framingham, MA, USA) equipped with electrospray ionization (ESI). Quantitative analysis of fucoxanthin and fucoxanthinol were also performed by LC-MS/MS. Detection was performed in positive ion mode under the following conditions: curtain gas at 30.0 L/h, collision gas medium, ion spray voltage at 5500 V, 550 °C, and ion source gases 1 and 2 both at 40 L/h. ESI-MS/MS parameters are shown in Table 1. The total chromatographic time was 7.0 min and retention times of fucoxanthin, fucoxanthinol, and IS were 3.76, 2.42, and 4.12 min, respectively. AB Analyst 1.6.1 software (ABSCIEX, Framingham, MA, USA) was used for system control and data acquisition.

### 4.3. Stock and Working Solutions

Individual standard stock solutions of fucoxanthin, fucoxanthinol, and IS (72, 60, and 47 μg/mL, respectively) were prepared in methanol. These stock solutions were then serially diluted with methanol to provide standard working solutions in the concentration range of 1.53–720 and 1.17–600 ng/mL for fucoxanthin and fucoxanthinol, respectively. All solutions were stored at 4 °C and brought to room temperature before use.

### 4.4. Calibration Standard Curves and QC Samples

Calibration standard (CS) curves were prepared by spiking 20 μL of the appropriate analyte working solution into 100 μL of blank rat plasma. The effective concentrations were 1.53, 3.06, 6.12, 12.25, 24.5, 45.0, 90.0, 180.0, 360, and 720 ng/mL for fucoxanthin and 1.17, 2.35, 4.7, 9.4, 18.8, 37.5, 75, 150, 300, and 600 ng/mL for fucoxanthinol. QC samples were prepared as a single batch for each concentration at 12.25, 90, and 360 ng/mL for fucoxanthin and 9.4, 75, and 300 ng/mL for fucoxanthinol, then divided into aliquots, and stored at 4 °C until use. The IS working solution of 235 ng/mL was diluted from stock solution as needed. Spiked rat plasma samples, serving as QCs, were processed following the sample procedure as for unknown samples.

### 4.5. Plasma Sample Preparation

To 100 μL of plasma sample (blank, spiked, or pharmacokinetics plasma sample) in a 1.5 mL polypropylene tube, 20 μL of IS working solution and 680 μL of methanol were added and mixed by vortexing for 1 min. After centrifugation at 25,000 × *g* for 10 min, the clear supernatant was filtered through a membrane (0.22-μmpore size), and injected into the LC-MS/MS system. When fucoxanthin and fucoxanthinol concentrations in rat plasma were greater than the calibration curve range, appropriate dilutions were applied to a plasma sample using blank rat plasma before further sample processing.

### 4.6. Method Validation

Assay validation was performed according to the currently accepted FDA prescription and per guidelines of the International Conference on Harmonization of Technical Requirements for Registration of Pharmaceuticals for Human Use. Selectivity was evaluated by comparing the selected ion recording (SIR) chromatograms of six different batches of blank plasma obtained from six rat subjects with those of corresponding spiked plasma containing fucoxanthin, fucoxanthinol, and IS, and a plasma sample obtained 0.5 h after an i.v. fucoxanthin dose. Each blank plasma sample was processed through the proposed extraction procedure and tested to ensure no rat plasma interference with the analyte.

LLOQ and LOD were defined as 10 and three times the S/N ratio, respectively. The lowest concentration on a calibration curve was accepted as the LLOQ if the analyte response was at least 10 times greater than that of blank plasma sample.

Linearity was assessed by plotting calibration curves in rat plasma induplicate in three separate runs. The calibration curves of fucoxanthin and fucoxanthinol were fitted by a linear, weighted (1/x^2^) least squares regression method through measurement of peak area ratios of the analytes to the IS. The LLOQ, defined as the lowest calibration curve concentration at which both precision and accuracy were less than or equal to a deviation of 20%, were evaluated by analyzing analyte-spiked plasma samples that were prepared as six replicates.

Accuracy and precision of the method were evaluated using QC samples at four concentrations (LLOQ and low, medium, and high QC) that were analyzed in six replicates on the same day and on three different days. Each run consisted of two calibration curves and six replicates of each concentration. The assay accuracy was expressed in terms of relative error (RE, %) and precision by relative standard deviation (RSD, %).

Extraction recoveries of fucoxanthin and fucoxanthinol were determined at three QC concentrations. Recoveries were calculated by comparing analyte/IS peak area ratios for each analyte in spiked plasma samples with those of analytes in the plasma matrices by spiking extracted analyte-free plasma samples prior to chromatography. Matrix effects from endogenous substances present in extracted rat plasma might have caused ion signal suppression or enhancement. Matrix effects at three QC concentrations (12.25, 90, and 360 ng/mL for fucoxanthin and 9.4, 75, and 300 ng/mL for fucoxanthinol) were measured by comparing peak responses of samples spiked post-extraction (A) with that of pure standard solution containing equivalent amounts of the two compounds (B). The ratio (A/B × 100%) was used to evaluate the matrix effect, with the extraction recovery and matrix effect of IS also simultaneously evaluated using the same method.

### 4.7. Stability

The stability of fucoxanthin and fucoxanthinol in rat plasma was assessed by analyzing replicates (*n* = 6) of three QC concentrations during sample storage and processing procedures. The freeze-thaw stability was determined through three freeze-thaw cycles. All stability testing of QC samples were determined using calibration curves of freshly prepared standards.

### 4.8. Pharmacokinetic and Bioavailability Studies

Male rats (ICR, 200 ± 20 g) were obtained from the Laboratory Animal Center of Xiamen University (Xiamen, China). Animal handling procedures were according to standard operating procedure approved by the institutional animal care and use committee. All rats were dosed following overnight fasting except for water *ad libitum*.

For pharmacokinetic studies, 12 male rats were randomly divided into two groups. Rats in Group 1 were administered i.v. with 2 mg/kg body wt of fucoxanthin. Serial blood samples (~0.3 mL) were collected in heparinized tubes via the jugular vein before and at time points of 0.08, 0.25, 0.5, 0.75, 1.0, 1.5, 2.0, 4.0, 6.0, 8.0, 12.0, 18.0, and 24.0 h after administration. In Group 2, each rat received the administration i.g. with 65 mg/kg body wt of fucoxanthin. Blood samples were collected before and at the time points of 0.5, 1.0, 2.0, 4.0, 6.0, 8.0, 10.0, 12.0, 14.0, 16.0, 24.0, 30.0, 36.0, and 48.0 h. Plasma was separated and stored frozen at −86 °C until analysis.

The following main pharmacokinetic parameters were analyzed using the non-compartmental pharmacokinetics data analysis software of PK solution 2™ (Summit Research Service, Montrose, CO, USA): maximum concentration (C_max_), time-to-maximum concentration (T_max_), half-life (*t*_1/2_), total body clearance (CL), steady state apparent volume of distribution (V_d_), area under curve from zero to the last measurable plasma concentration point (AUC_0__–_*_t_*, *t* = 24 h for i.v. and t = 48 h for i.g. administrations), and area under the plasma concentration–time curve from zero to time infinity (AUC_0–∞_). Oral bioavailability is defined as the fraction of unchanged drug reaching the systemic circulation following administration by the i.g. route. Absolute oral bioavailability of a drug is generally measured by comparing the respective AUC_(0__–__∞)_ after i.g. and i.v. administrations, according to the following equation:

$$F=\frac{\text{AUCi}.\text{g}./\text{Dosei}.\text{g}.}{\text{AUCi}.\text{v}/\text{Dosei}.\text{v}.}$$

## 5. Conclusions

A rapid, reliable, and sensitive LC-MS/MS method for simultaneous determination of fucoxanthin and fucoxanthinol, its major metabolite, in rat plasma is described for the first time. Both compounds were identified in plasma after i.v. and i.g. administrations. This method was successfully applied to a pharmacokinetic study of fucoxanthin and fucoxanthinol in rats.
